# Staffing Patterns of Non-ACGME Fellowships with 4-Year Residency Programs: A National Survey

**DOI:** 10.5811/westjem.18454

**Published:** 2024-02-28

**Authors:** David A. Haidar, Laura R. Hopson, Ryan V. Tucker, Rob D. Huang, Jessica Koehler, Nik Theyyunni, Nicole Klekowski, Christopher M. Fung

**Affiliations:** * University of Michigan, Department of Emergency Medicine, Ann Arbor, Michigan; † University of Colorado, Department of Emergency Medicine, Aurora, Colorado

## Abstract

**Introduction:**

Emergency medicine (EM) is one of few specialties with variable training lengths. Hiring a three-year graduate to continue fellowship training in a department that supports a four-year residency program can lead to conflicts around resident supervision. We sought to understand hiring and clinical supervision, or staffing, patterns of non-Accreditation Council for Graduate Medical Education (ACGME) fellowships hosted at institutions supporting four-year residency programs.

**Methods:**

We performed a web-based, cross-sectional survey of non-ACGME fellowship directors (FD) hosted at institutions supporting four-year EM residency programs. We calculated descriptive statistics. Our primary outcome was the proportion of programs with four-year EM residencies that hire non-ACGME fellows graduating from three-year EM residencies.

**Results:**

Of 119 eligible FDs, 88 (74%) completed the survey. Seventy FDs (80%) indicated that they hire graduates of three-year residencies. Fifty-six (80%) indicated that three-year graduates supervise residents. Most FDs (74%) indicated no additional requirements exist to supervise residents outside of being hired as faculty. The FDs cited department policy, concerns about quality and length of training, and resident complaints as reasons for not hiring three-year graduates. A majority (10/18, 56%) noted that not hiring fellows from three-year programs negatively impacts recruitment and gives them access to a smaller applicant pool.

**Conclusion:**

Most non-ACGME fellowships at institutions with four-year EM programs recruit three-year graduates and allow them to supervise residents. This survey provides programs information on how comparable fellowships recruit and staff their departments, which may inform policies that fit the needs of their learners, the fellowship, and the department.

## INTRODUCTION

Emergency medicine (EM) is one of few specialties in the United States with variable training lengths.^1^ ^,^ ^2^ Most residencies implement a three-year model, while only 20% implement a four-year model.^3^ ^–^ ^5^ There is little data to support either training length.^3^ ^–^ ^7^ Some argue that four-year graduates have more time to gain confidence, develop procedural skills, develop academic interests, and gain experience supervising learners. Advocates of three-year programs argue that an extra year as faculty would provide these same experiences.^1^ ^,^ ^8^ ^,^ ^9^ These personal biases may impact recruitment and hiring of three-year graduates at institutions supporting four-year residency programs.^6^ ^,^ ^7^

When an institution hosting a four-year residency hires a three-year graduate into fellowship training, this can lead to conflicts around clinical supervision, or staffing, of residents related to perceptions of seniority and quality of training.^1^ ^,^ ^9^ There are currently no best practices or guidelines to inform programs on how to address this situation. The situation is further complicated as non-Accreditation Council for Medical Education (ACGME) fellowships frequently lack uniform rules that govern recruitment, program requirements, and clinical responsibilities.^10^ No studies currently evaluate the prevalence of these issues or examine variability in recruitment, hiring, and clinical responsibilities of trainees at non-ACGME fellowships. In this study, we sought to understand the hiring and staffing patterns of non-ACGME fellowships hosted at institutions with four-year EM residency programs.

## METHODS

### Study Design and Participants

This was a cross-sectional survey of fellowship directors (FD) of non-ACGME fellowships hosted at institutions supporting a four-year EM residency program. We conducted the survey between January–April 2023. This study was deemed exempt by our institutional review board (HUM00221519). In November 2022, we generated a list of 54 four-year EM residency programs from the Emergency Medicine Residents’ Association (EMRA) Match roster and Electronic Residency Application Service directory.^11^ ^–^ ^13^ We identified non-ACGME fellowships offered using each program’s webpage, the Society for Academic Emergency Medicine Fellowship Directory, and the Society for Clinical Ultrasound Fellowships directory.^14^ ^,^ ^15^

### Survey Development and Distribution

We developed the survey based on Panacek’s general survey principles, literature review, and expert opinion to provide content validity evidence.^6^ ^,^ ^16^ ^–^ ^18^ All authors have experience developing survey studies, and the group (including four current or former FDs) iteratively piloted and revised the survey for optimal phrasing, survey length, functionality, and appropriate mix of suggested and open-ended responses, which provided content and response process validity evidence.^18^ We used Qualtrics (Qualtrics XM, Provo, UT), a web-based survey platform, to distribute the survey via email with a personalized link for each FD to collect and analyze the data. We sent weekly reminders to FDs’ institutional emails, with an option to decline participation, for eight weeks. We then sent personalized weekly reminder emails for an additional four weeks. We collected individual responses to the survey anonymously.

### Outcomes and Data Analysis

We asked FDs to report their fellowship type, years in current role, and demographic data such as number of clinical sites, program environment (academic, county, community, etc), and geographic location. Our primary outcome was the proportion of programs affiliated with four-year EM residencies that hire non-ACGME fellows graduating from three-year EM residencies. We also asked clarifying questions to better understand their staffing model, and recruiting, hiring, and clinical oversight policies. The survey included space for comments so that the FDs could provide context to their answers, but we did not analyze these for themes. The full survey is available in Appendix A1. We analyzed the data using Excel 365 (Microsoft Corporation, Redmond, WA) to generate descriptive statistics and analysis. We assessed the association between categorical variables using the Fisher exact test. We did not calculate an a priori sample-size estimate as we attempted to capture a 100% response rate.

## RESULTS

Of 54 four-year EM residencies in the US, 32 institutions offered at least one non-ACGME fellowship with a total of 128 fellowships identified (median 3.5; range 1–10). We received 88 responses after excluding nine opt-outs and one blank response (88/119) for a response rate of 73.9%. Program and FD characteristics are listed in the Table. Free text responses are included in Appendix A2.

**Table.** Demographic details of the fellowships represented in our survey of fellowship directors of non-ACGME fellowship programs.

Demographics	Number of responses (%)
Fellowship type	
Admin/operations	14 (15%)
Cardiology and resuscitation	1 (1%)
Climate and health policy	1 (1%)
Digital health	1 (1%)
Disaster medicine	3 (3%)
Global health/international medicine	7 (7%)
Health humanities	1 (1%)
Health policy	1 (1%)
Medical education	18 (19%)
Neurologic emergencies	1 (1%)
Pediatric ultrasound	1 (1%)
Physician wellness	1 (1%)
Research	9 (9%)
Simulation	5 (5%)
Social medicine	3 (3%)
Ultrasound	22 (23%)
Wilderness medicine	3 (3%)
Program region	
Central (IL, IN, IA, KS, MI, MN, MO, NE, OH, WI)	13 (15%)
Northeast (CT, DC, DE, MA, MD, ME, NH, NJ, NY, PA, RI, VT)	45 (51%)
Southern (AL, AR, FL, GA, KY, LA, MS, NC, OK, PR, SC, TN, TX, VA, WV)	0 (0%)
Western (AZ, CA, CO, NM, NV, OR, UT, WA)	30 (34%)
Category of primary residency site*	
Academic (university based)	81 (82%)
Community	0 (0%)
County	15 (15%)
Other	3 (3%)
Category of non-ACGME fellow’s primary clinical site*	
Academic (university based)	74 (46%)
Community	46 (29%)
County	23 (14%)
Other	17 (11%)
Number of clinical sites non-ACGME fellows clinically staff	
1	22 (25%)
2	33 (38%)
3	26 (30%)
4	6 (7%)

* Respondents could select more than one type of clinical site.

Of the 88 responses, 70 FDs (80%) reported hiring graduates of three-year EM programs for their respective fellowships. Fifty-six FDs (80%) who accept three-year graduates indicated that their fellows can supervise EM residents. We found variation in who fellows could supervise. The most common policy (40%) was that fellows can supervise EM postgraduate-year (PGY)-3 residents and below. Most FDs (74%) indicated that they had no additional requirements to supervise residents outside of being hired on as faculty. Full survey results appear in the Figure.

**Figure.**
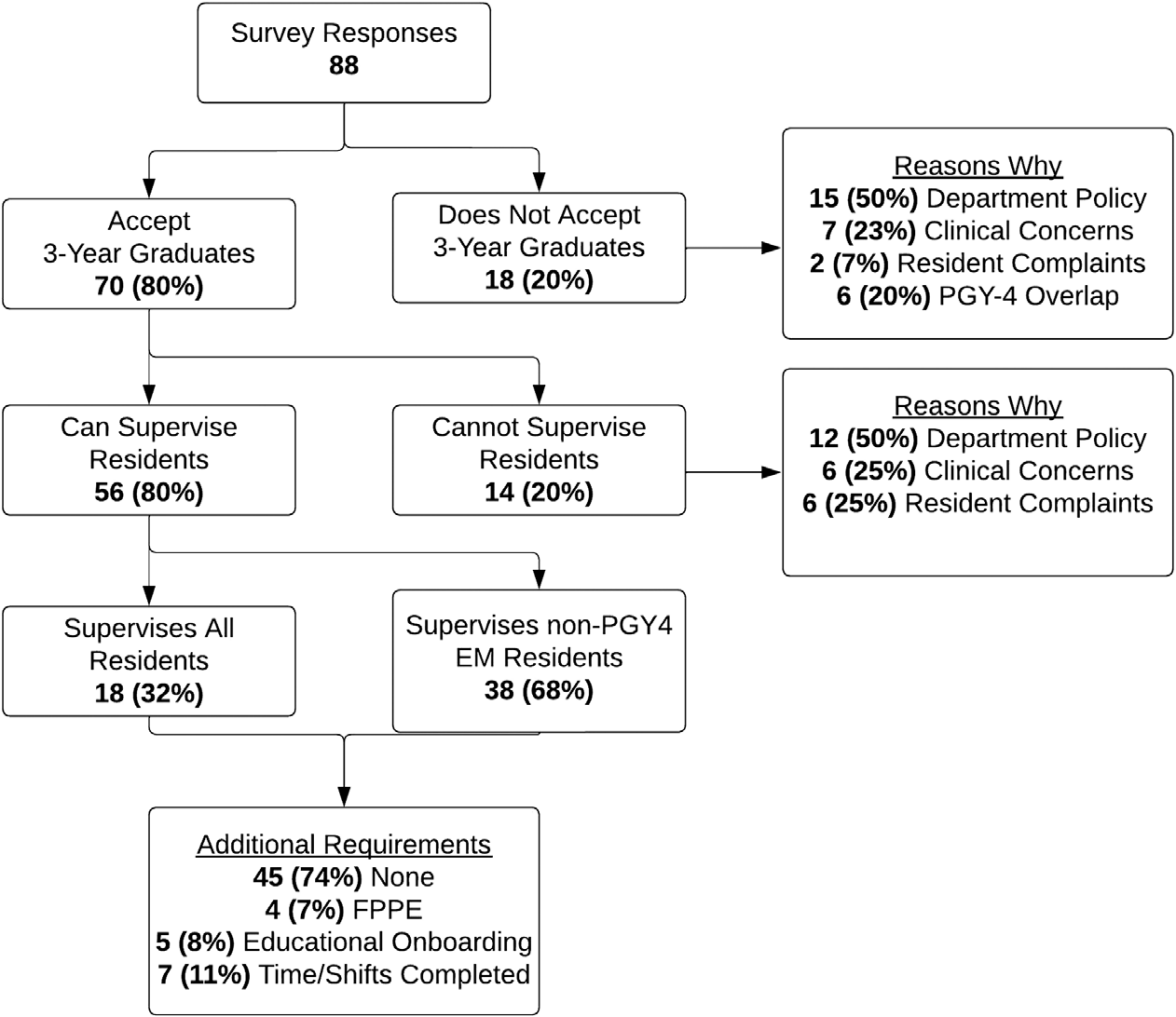
Flow diagram detailing the survey response hierarchy of fellowship directors of non-Accreditation Council for Graduate Medical Education fellowships regarding clinical supervision patterns for 3-year emergency medicine (EM) residency graduates at institutions with a 4-year EM program. *EM*, emergency medicine; *PGY*, postgraduate year; *FPPE*, focused professional practice evaluation.

Programs with multiple clinical sites are more likely to hire three-year graduates. Ten of 23 programs (57%) with one clinical staffing site hired three-year graduates compared to 88% (57/65) of sites with two or more clinical sites (*P* < 0.001). The FDs reported the implementation of various strategies to mitigate potential conflicts. One program hosts a joint fellowship curriculum for their fellows, which incorporates instruction on bedside teaching, giving feedback, and teaching various skills. Other FDs reported that their programs prevented their fellows from staffing in high acuity areas or delay working with residents.

Twenty-seven FDs (50%) cited department policy as the reason for their hiring and staffing policies. Selected comments from other FDs included concerns about quality and length of training and resident complaints. Others reported their clinical environment was not conducive to separating fellows from residents. Seven FDs reported wanting to avoid PGY-4 fellows staffing PGY-4 residents. One FD indicated that “because we are a 4-year program, we want to acknowledge to our residents that 4 years is what we think is required for graduation.”

Among the programs not hiring fellows from three-year programs, 56% (10/18) of FDs noted that this policy negatively impacted their fellowship recruitment and gave them access to a smaller pool of applicants.

## DISCUSSION

To our knowledge this study is the first to describe staffing patterns of non-ACGME fellowships hosted at institutions with four-year EM residencies. Most of the FDs we surveyed hire three-year graduates as fellows, and most programs permit three-year graduates to staff residents with no additional requirements beyond being hired. We also identified potential negative impacts on fellowships as they restrict their applicant pool. One FD indicated that their fellowship was mostly going unfilled due to their recruitment policy. Another indicated that the financial sacrifice of a four- vs three-year residency may unintentionally favor recruitment of those without financial need or burden, especially since the debt load of EM applicants is reportedly higher than for other medical specialties.^1^

Some programs offer their fellows alternative clinical sites – such as Veterans Affairs hospitals, freestanding EDs, or urgent cares. By staffing multiple locations, non-ACGME fellows can work without a resident presence. This flexibility allows programs to hire three-year graduates and permits fellows to interface with residents academically without having to supervise them clinically. This allows for a training environment conducive to the needs of all learners’ growth and development.

The FDs cited clinical concerns and department policy as the main reasons for their staffing and hiring policies. There is a lack of objective data that four-year graduates outperform three-year graduates clinically or on the qualifying written board exam, suggesting that this may be rooted in bias.^1^ ^,^ ^6^ ^,^ ^7^ In the absence of robust data to support the clinical capabilities of trainees from either three- or four-year programs, the principles of competency-based medical education (CBME) may offer solutions.^19^ The principles of CBME require demonstration of competency and decouple attainment of competency from time-in-training.^19^ The use of CBME to determine readiness for unsupervised practice through a process known as “promotion in place” has been piloted by some residency programs and may be a useful model to replicate in determining fellow readiness for staffing, regardless of PGY status.^19^ ^,^ ^20^ If we remove the focus from time-bounded training and focus on demonstrated skill acquisition, programs may design processes to onboard three-year graduates by focusing on developing and assessing appropriate skills for supervision of trainees.

Future studies could explore who sets departmental policies regarding fellow staffing, evaluate fellow and resident perceptions of staffing policies, and compare career outcomes of fellows working in various staffing environments.

## LIMITATIONS

We may not have captured all non-ACGME fellowships at four-year institutions. We did not identify fellowship directories besides ultrasound, which may have led to sampling bias. We attempted to mitigate this by searching specific program websites for listed fellowships. The FDs who did not participate in our study may represent a unique population with different hiring and staffing patterns. We did not identify non-ACGME fellowships hosted at four-year EM programs in the southern US, nor did we receive responses from primarily community EM programs, which could also have biased our results. We did not survey ACGME-accredited fellowships, as fellows vary in the way they “maintain their primary Board skills.”^21^ Some ACGME fellowships (eg, critical care, emergency medical services) do not require minimum clinical hours in the emergency department, which leads to a qualitatively different experience from non-ACGME fellowships, where fellows are appointed as clinical faculty.^2^ ^,^ ^21^ ^,^ ^22^

## CONCLUSION

Our results indicate that most non-ACGME fellowships hosted at institutions with four-year EM programs recruit graduates of three-year programs and allow them to supervise residents. This survey data provides program information on how comparable fellowship programs recruit and staff their departments, which may inform policies that fit the needs of their learners.

## Supplementary Information



