# Supplementary material for: Staffing Patterns of Non-ACGME Fellowships with 4-Year Residency Programs: A National Survey
Source: West J Emerg Med. 2024 Feb 28;25(2):175–80. doi: 10.5811/westjem.18454 (PMC11000558; doi:10.5811/westjem.18454)
Supplement: Supplementary file 1 [file wjem-25-175-s001.docx]

**Four Year Residency Staffing Models - FINAL DRAFT**

**Q1 - What is your job title? (Select All That Apply)**

| # | Answer |
| --- | --- |
| 2 | Admin/Operations Fellowship Director |
| 3 | Research Fellowship Director |
| 4 | Medical Education Fellowship Director |
| 5 | Ultrasound Fellowship Director |
| 6 | Wilderness Medicine Fellowship Director |
| 7 | Other non-ACGME Fellowship Director (Please Specify): |
| 8 | Other (Please Specify): |
| 10 | Simulation Fellowship Director |
| 11 | Global Health/International Medicine Fellowship Director |
| 12 | Social Medicine Fellowship Director |

**Q2 - How many years have you been in this role?**

**Q3 - What geographic region is your program located in?**

| # | Answer |
| --- | --- |
| 1 | Central (IL, IN, IA, KS, MI, MN, MO, NE, OH, WI) |
| 2 | Northeast (CT, DC, DE, MA, MD, ME, NH, NJ, NY, PA, RI, VT) |
| 3 | Southern (AL, AR, FL, GA, KY, LA, MS, NC, OK, PR, SC, TN, TX, VA, WV) |
| 4 | Western (AZ, CA, CO, NM, NV, OR, UT, WA) |

**Q4 - How would you describe your residency program? (Select All That Apply)**

| # | Answer |
| --- | --- |
| 1 | Academic (University based) |
| 2 | Community |
| 3 | County |
| 9 | Other: |

**Q5 - How many different sites do your non-ACGME fellows clinically staff?**

**Q6 - How would you describe the site(s) that your fellows staff? (Select All That Apply)**

| # | Answer |
| --- | --- |
| 1 | Academic (University based) |
| 2 | Community |
| 3 | County |
| 4 | Other: |
|  | |

**Q7 - Do you hire/match graduates of 3-year Emergency Medicine residencies for your specific fellowship program?**

| # | Answer |
| --- | --- |
| 1 | Yes |
| 4 | No |
| 5 | Other: |

**Q8 - Do you allow non-ACGME fellows who graduate from 3-year programs to staff residents in the Emergency Department?**

| # | Answer |
| --- | --- |
| 1 | Yes, only off-service residents |
| 2 | Yes, all EM residents |
| 5 | Yes, EM PGY-3 and below |
| 6 | Yes, EM PGY-2 and below |
| 7 | Yes, EM PGY-1s |
| 8 | Other: |
| 11 | No |
| 13 | Yes, all residents (EM & off-service) |

**Q9 - Are there specific requirements that your fellows must meet to staff EM residents? (Select All That Apply)**

| # | Answer |
| --- | --- |
| 1 | No additional requirements beyond being hired as a fellow |
| 2 | Yes, must complete clinical Focused Professional Practice Evaluation (FPPE) requirements |
| 3 | Yes, must complete education-specific onboarding requirements |
| 4 | Yes, must complete a certain amount of time or shifts |
| 5 | Other: |

**Q10 - Please provide additional details of requirements your fellows must meet to staff residents.**

**Q11a - Why do you not allow fellows who graduate from 3-year programs to staff residents? (Select All That Apply)**

| # | Answer |
| --- | --- |
| 1 | Institutional Policy |
| 2 | Department Policy |
| 3 | Clinical Concerns About Quality or Length of Training |
| 4 | Clinical Concerns Due to EM Resident Complaints |
| 5 | Fellow Preference |
| 6 | Other (Please Clarify) |

**Q11b - Please provide any clarifying comments:**

**Q12a - Why do you not match/hire fellows who graduate from 3-year programs? (Select All That Apply)**

| # | Answer |
| --- | --- |
| 1 | Institutional Policy |
| 2 | Department Policy |
| 3 | Clinical Concerns About Quality/Length of Training |
| 4 | Clinical Concerns Due to Resident Complaints/Feedback |
| 5 | Other (Please Clarify) |
|  |  |

**Q12b - Do you feel like this has impacted your recruiting? Please provide clarification below if able to.**

| # | Answer |
| --- | --- |
| 1 | Yes, positively |
| 2 | Yes, negatively |
| 3 | No |
| 4 | Other: |

**Q12c - Please provide any clarifying comments:**

**Q13 - Please provide clarifying comments or questions if you have any.**
