# Supplementary material for: Staffing Patterns of Non-ACGME Fellowships with 4-Year Residency Programs: A National Survey
Source: West J Emerg Med. 2024 Feb 28;25(2):175–80. doi: 10.5811/westjem.18454 (PMC11000558; doi:10.5811/westjem.18454)
Supplement: Supplementary file 2 [file wjem-25-175-s002.docx]

**Q10. Please provide additional details of requirements your fellows must meet to staff residents.**

- On-boarding; shadow shifts
- they cannot staff R-4s until halfway through the year
- Cannot work in the high acuity area of the ED, or supervise PGY4 residents, for the first year.
- One site- can work immediately with PAs Other site with residents- can work in fast track or side without PGY3 until 6 months in and then can supervise PGY4
- Our emergency department staffing is such that we can schedule fellows so that they never supervise PGY-4s, but our fellows are subject all of the same faculty requirements as any other attending, which includes quantitative ongoing data review, initial FPPE, and a very deliberate and specific 20 hour onboarding program

**Q11ac. Why Do You Not Allow Fellow Who Graduate From 3-Year Programs to Staff Residents? Please Provide Any Clarifying Comments**

- Our fellows staff family medicine and other off service residents rotating through our various departments. Second year fellows are allowed to staff EM residents
- The fellows do not work with our residents at our community site as we do not have residents there. They do not staff their cases at the VA hospital because our PGY4 residents do not rotate there. There is no policy since there is no potential for that interaction currently.
- no written policy exists, but it is the accepted policy of the department
- Fellows that have completed 4 years of training (i.e. may have done surgery internship then switched to a 3 year EM program ARE allowed to supervise residents). While pgy3 graduates do now work at our main academic center, they do sometimes supervise residents at the community hospital location.
- We place graduates from three year programs at a site where they do not staff patients with residents.
- PGY4s supervising PGY4s can get messy.
- We do not allow EM PGY4s to staff so it would be inconsistent.

**Q12ac. Why Do You Not Match/Hire Fellows Who Graduate From 3-Year Programs? Please Provide Any Clarifying Comments**

- Because we are a 4 year program and want to acknowledge to our residents that 4 years is what we think is required for graduation.
- We're a 4 year program so it would mean our PGY-4 fellow will be supervising our PGY-4's.
- ACGME requirements that PGY 4 resident cannot be supervised by an attending or fellow at the same post-graduate year (e.g they could not supervise our PGY 4 residents and therefore would not be able to work our Acute area which is critical for education of residents and the fellows' clinical experience).
- Cannot separate from PGY4s in our staffing models, did not want fellows supervising residents of same PGY
- We have a 4 year residency and it creates difficulty with graduates/fellows from 3 year programs to supervise our PGY-4 residents. We do not have an area or pod where only junior residents work. We feel that supervision of residents is an essential part of being a medical education fellow.
- All of the shifts that an attending could work might be paired with a PGY 3 or 4 senior resident and there is no shift that a PGY 4 resident wouldn't work. We do not feel it is appropriate for a PGY 4 attending to supervise a PGY 4 resident

**Q12b. Do You Feel Like This Has Impacted Your Recruiting? Please Provide Clarification Below If Able To.**

- Definitely have access to less applicants
- Lower pool
- Many of our fellowships are now going unfilled.
- Smaller pool of applicants
- The pool of applicants from 4 year programs or 3-year + work, is quite small. Every year we get many requests from 3-year applicants but cannotmaccept them.
- Social EM is supposed to recognize the influence of our social needs on our decisions. Financial sacrifice of a 4 year residency followed by a 2 year fellowship is significant and favors recruitment of those without financial need or burden. It seems like in Social EM we should want those with a passion for health equity fueled by their own experience and we should not limit opportunities for these graduates.

**Q13. Please Provide Clarifying Comments or Questions in General if You Have Any.**

- Our residents who graduate from 3-year residencies work at sites where our PGY4 residents do not work. They do not interact.
- I would like to present my Department with some feasible strategies to recruit 3-year graduates for fellowship because this has been successful at other 4-year residency institutions. But so far there has been a fair amount of resistance.
- We have a wide range of fellowships and to date we only accept graduates from 4 year programs due to conflicts with scheduling a graduate of a 3-year program and supervising PGY-4 residents. We have an exception in our research fellowship because they can work in multiple other sites where they do not supervise residents and they work conference shifts when no learners are present.
- All of our fellows are ABEM approved EM Residency graduates.
- 3 year grads primarily work at our community hybrid site during their first year of fellowship to minimize direct supervision of 4th year residents. However, there is no hard rule about who they can and cannot supervise. They are board eligible attendings and have the responsibilities of an attending when working clinically.
- Currently working to develop a competency based process by which fellows who have graduated from a PGY-3 format program can develop and demonstrate supervisory/instructional skills and earn the privilege of staffing junior residents.
- We have multiple cores in our ED and the PGY4 EM residents work out of only 1 of those cores. So, during their first year of fellowship a fellow from a 3 year program isn't scheduled for any shifts in that core. This restriction is lifted during their 2nd year. Overall, this has minimal impact on their attending-ship as we have many cores and many shift options.
- We have had wonderful experiences with graduates of both three-year and four-year residency programs, and our philosophy has been that we evaluate applicants on their individual merits, not on the basis of the length of their training program.
- While fellows are able to work with all PGY levels the reality is that as a a combined academic/university hospital and county hospital program, the PGY 3 and 4 levels are split between the systems with PGY3 serving as seniors most of the time where our fellows work.
